# Surgical management of a Salter-Harris type I proximal physeal fracture of the tibia in a foal: a case report

**DOI:** 10.1186/s12917-022-03285-6

**Published:** 2022-05-16

**Authors:** Ramin Mazaheri-Khameneh, Saeed Azizi, Farshid Davoodi, Mohammad Mahdi Gooran

**Affiliations:** Department of Surgery and Diagnostic Imaging, Faculty of Veterinary Medicine, Urmia University, Urmia, Iran

**Keywords:** Proximal tibial fracture, Salter-Harris type I, Tibial tuberosity fracture, T-plate fixation, Lag screwing, Foal

## Abstract

**Background:**

One of the traumatic fractures occurring in the hindlimb of the foals is physeal fractures of the tibia. The most common type of proximal tibial fracture in foals is reported to be Salter-Harris type II.

**Case presentation:**

This case report describes the history, clinical signs, radiographic findings, and surgical management of a proximal physeal fracture of the tibia due to the kick trauma in a 2-weeks-old Kurdish female foal, with signs of left pelvic limb lameness, valgus deviation with non-weight-bearing, non-mobility in stifle region and crepitation in the affected area. In this case, radiography was utilized to confirm the fracture and detect the exact location of the fracture fragments. The Salter-Harris type I accompanied by tibial tuberosity fracture was diagnosed. In this case, a size 4.5 mm seven-hole, T-plate, and cortical bone screws were employed to fix the physeal fracture, and a cortical bone screw was utilized to fix the tibial tuberosity in the normal position. Case follow-up during two months showed no significant complications, and the patient was fully recovered (no lameness anymore).

**Conclusions:**

To our knowledge, this is the first report of Salter-Harris type I fracture in proximal tibia accompanied by tibial tuberosity fracture in a foal treated by a T-plate implant. A cortical screw in lag fashion for tibial tuberosity was utilized in this case for the first time, and the results were satisfying. T-Plate fixation can be recommended as an effective surgical treatment for proximal tibial fractures in foals.

**Supplementary Information:**

The online version contains supplementary material available at 10.1186/s12917-022-03285-6.

## Background

Physis or growth plate is a cartilaginous plate that contributes to the longitudinal growth of the long bones. Owing to the cartilaginous structure of the physis, it is a weak point in the bone and can be easily damaged in growing animals [1]. Of the physeal fractures in horses, only 10% belong to the tibia [2]. Physeal injuries of the tibia in foals mainly include proximal physis [3]. The most prevalent fracture of the tibia in foals is known to be a Salter-Harris type II with a metaphyseal segment involving up to one-third of the physeal surface [4]. One of the most common causes of equine fractures is being kicked by another horse, and fractures most commonly involve limbs [5]. Severe lameness is evident in the proximal growth plate and tibial tuberosity fractures, but soft tissue trauma is rarely observed in these fractures, and in most cases, the skin of the injured region is intact [6]. Moreover, valgus deformity of the hindlimb distal to the fracture site is observed in proximal physeal, and diaphyseal tibial fractures [6]. Manipulation of the limb reveals crepitation and instability of the fracture region [7]. Radiography assessment confirms the diagnosis and helps to find and identify precisely the location and severity of bone fracture [6]. Salter-Harris Type I physeal fracture in the proximal aspect of the tibia in foal has not been reported previously. The present study describes the history, clinical signs, radiographic evaluations, and surgical management in a two-week-old Kurdish foal.

## Case presentation

**Fig. 1:**
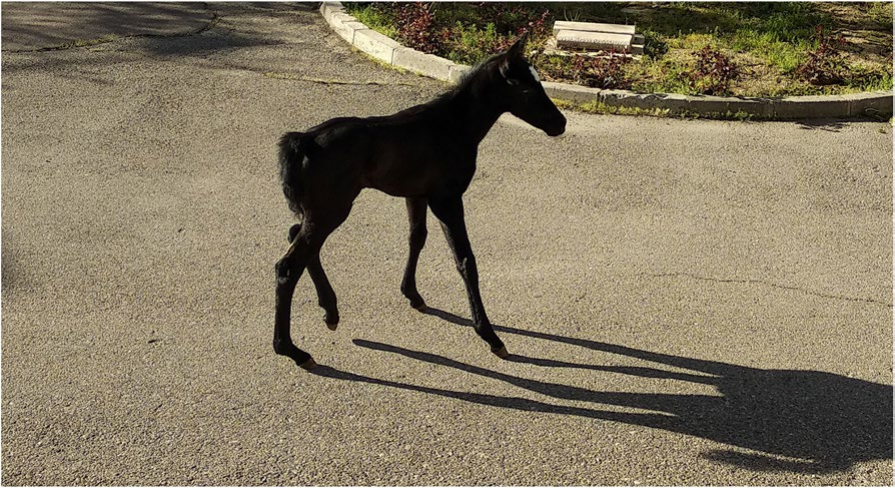
Referred foal with signs of non-weight bearing lameness of the left hindlimb

**Fig. 2:**
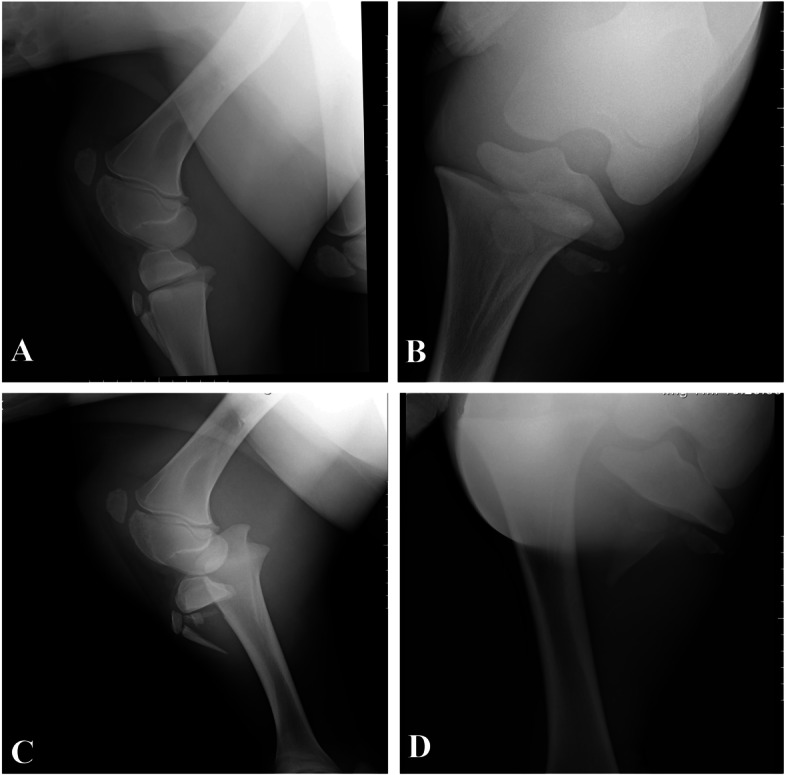
**A** and **B** lateral and craniocaudal radiographs of the left hindlimb on the first day of the referral, respectively. **C** and **D** lateral and craniocaudal radiographs of the left hindlimb the day after first referral

A two-week-old, 70 kg, female Kurdish foal was referred to the Urmia University Veterinary Hospital with lameness and a history of kick trauma from her mother (Fig. 1). On presentation, the foal’s general condition was good. Physical examination revealed severe left pelvic limb lameness, valgus deviation with non-weight-bearing, non-mobility in the stifle region, and crepitation. No skin laceration was observed during physical examinations. The foal was sent to the radiology and the diagnostic imaging section of the veterinary hospital and radiographic examination was performed in two standard radiographic views (lateromedial and caudocranial). A Salter-Harris type I fracture of the proximal physis of the tibia with a dorsomedial displacement of the tibia was diagnosed (Fig. 2 A, B).

The next day, before surgical procedures, radiographs were made again. It was observed that fracture fragments were displaced more, overridden, and a tibial tuberosity fracture was now obvious (Fig. 2 C, D).

**Fig. 3:**
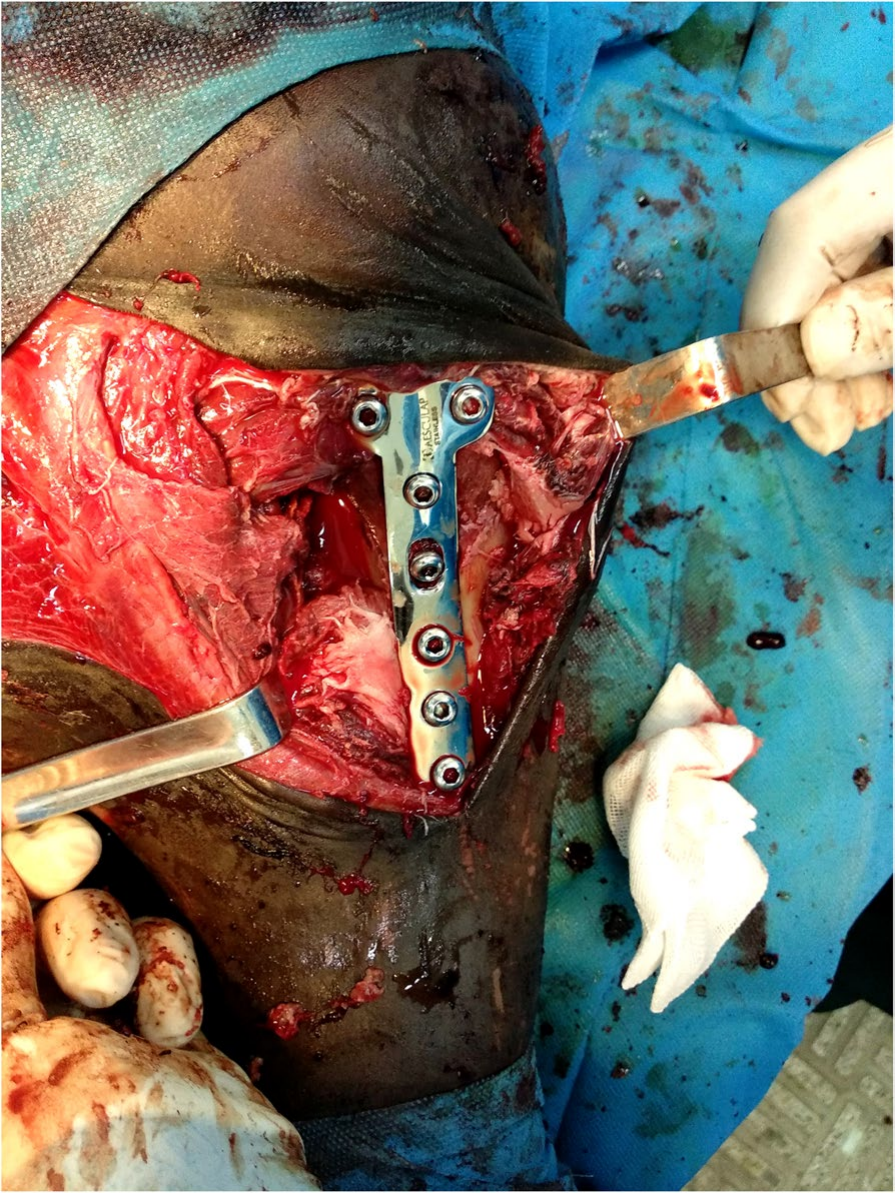
Intraoperative appearance of T plate after fixation with cortical screws

**Fig. 4:**
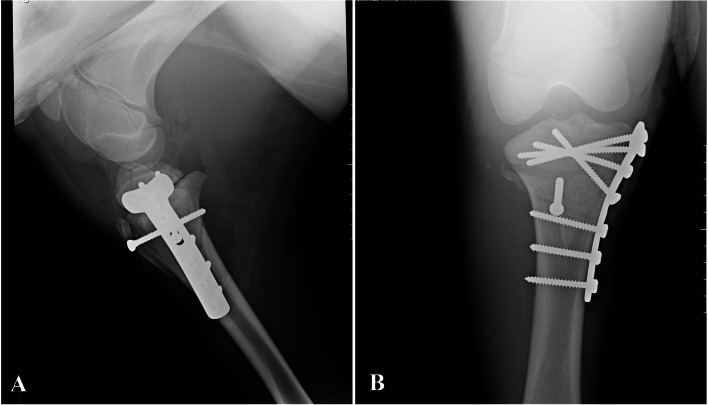
**A** and **B** lateromedial and craniocaudal views of the tibia after surgery and fixation with T plate

After premedication with midazolam (0.05 mg/kg, IV), induction was done using the combination of midazolam (0.05 mg/kg, IV) and ketamine-hydrochloride (2 mg/kg, IV). Preoxygenation was performed using a mask (4 L/min) simultaneously with induction of anesthesia. Subsequently, the patient was intubated, and anesthesia was maintained with isoflurane in oxygen. Cefazolin (20 mg/kg, IM) and gentamicin (6.6 mg/kg, IV) were used as prophylactic antibiotics. The foal was positioned in left lateral recumbency on the tilt table so that the affected limb was located down. The proximal aspect of the left pelvic limb was clipped and shaved around and prepared for routine aseptic surgery. The tibial medial approach was used for surgery so that a 10 cm skin incision along the medial aspect of the tibia was made. The incision was continued along with the subcutaneous tissue and superficial fascia. The soft tissue was elevated cranial and caudal, and the fracture site was exposed. The fracture was reduced by manual traction and rotation of the limb and temporarily maintained with pointed reduction forceps. For fixation of the proximal epiphysis of the tibia, a size 4.5 mm seven-hole, T-plate, and cortical bone screws (Four cortical screws with lengths of 7 cm in the proximal segment of fracture and three cortical screws with lengths of 4.6, 4.2, and 4.2 cm in the distal segment of the fracture, respectively) were utilized on the medial aspect of the tibia. Tibial tuberosity was fixed using a cortical bone screw with a length of 6 cm in lag fashion (Fig. 3). Eventually, the surgical site was closed routinely and secured with a stent bandage. Radiographs were made at the end of the surgical procedure, and it was observed that the fracture was completely reduced (Fig. 4). The foal recovered from anesthesia. Afterwards, post-operative treatment consisted on cefazolin (20 mg/kg, q 12 h, IM), gentamicin (6.6 mg/kg, q 24 h, IV) and flunixin meglumine (1.1 mg/kg, q 24 h, IV) for 5 days. Two days after surgery, the foal had relatively good weight-bearing on the left pelvic limb (Additional file 1). Following five days of hospitalization, the foal was discharged and necessary recommendations about wound care and movement restrictions were given to the foal owner. He was also advised to return to the hospital one month after surgery for re-evaluation.

**Fig. 5:**
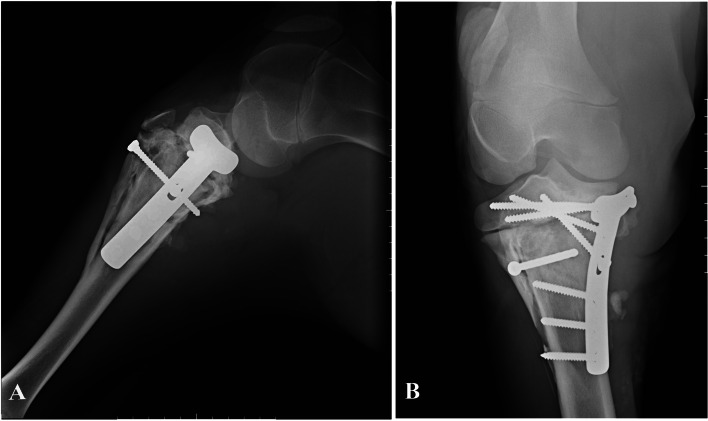
**A **and** B **lateromedial and craniocaudal radiographs of tibia two months following the operation. Callus formation is visible in the radiograph

**Fig. 6:**
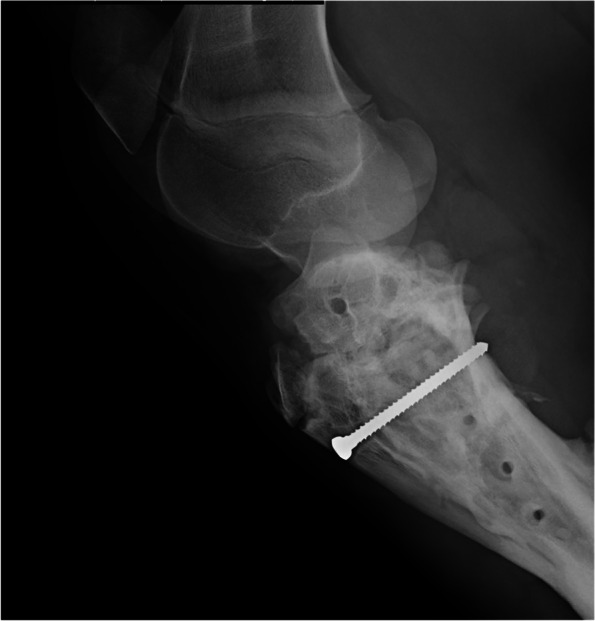
Lateral radiograph of the proximal tibia after surgical removal of the T-plate

One month after the surgery, a purulent fistula was observed at the surgical site. Moreover, some callus was seen in the radiographic assessment of the fracture site. The purulent fistula was controlled by flushing the area with normal saline 0.9%. Based on expected flora in this region, cefazolin (20 mg/kg, IM) and gentamicin (10 mg/kg, IV) were initially utilized until choice antibiotics were discerned due to the culture and sensitivity results. However, sensitivity results confirmed the chosen antibiotics, and the patient was discharged after three days of hospitalization. Medical treatment was continued until no more discharge was observed (7 days). Eight weeks after surgery, the foal was referred for the second control. No signs of lameness were observed, and the foal was able to bear weight on the affected limb. Surgical site infection was improved. Subsequently, radiographs were taken, and callus formation was confirmed. Signs of surgical site infection were not observed in the radiograph, and the bone was healed, which indicated the success of the surgery (Fig. 5). Ultimately, the plate was removed, and the tibial tuberosity screw was left in place (Fig. 6). The cortical screw was not removed because the tibial tuberosity fracture was not completely healed, and there were no signs of infection-related. Long-term follow-up of the foal after five months revealed normal growth, and no angular limb deformity was observed.

## Discussion and conclusion

Proximal physeal fractures in foals prevalently occur due to a kick from another horse. Mostly when the animal is weight-bearing on a hindlimb, and the kick happens from the lateral aspect [8]. In eventing horses, tibial tuberosity fractures occur following hitting a solid fence with the stifle during jumping. The most probable reason for fractures of the tibial tuberosity in non-eventing horses is direct kick injury. The stifle joint may be involved in the tibial tuberosity fractures, and the fracture fragment is usually displaced proximally [6]. In the present case, the proximal physeal fracture occurred due to the kick injury from the mother. On the first day of the referral, light displacement of the fracture segments was observed in the radiographs. Watkins JP and Sampson SN (2019) noted that external coaptation of the fractured limb before doing surgical procedures in the cases of proximal physeal fractures of the tibia is not advocated owing to the fracture configuration that does not cause an open injury [6]. Consequently in the present case, the owner was advised to decrease the foal’s activity to reduce further damages and displacement of the fragments [6]. On the second day of referral, radiographs were made before the surgery, and the distal fragment was completely displaced dorsomedial, and fracture of tibial tuberosity was now observed. Hence, in contrast to Watkins JP and Sampson SN (2019), external coaptation or immobilization may be recommended for proximal tibial fractures, not only to reduce the risk of an open fracture but also to reduce the risk of further displacement of the fracture as it happened in the present case. Lateral splinting is recommended for this kind of fracture before surgery. However, splinting could also be detrimental in some cases. Moreover, surgical interventions should not be delayed, and prompt intervention is critical to avoid further damages.

Various techniques are available for surgical treatment of proximal physeal fractures of the tibia. Cross pin fixation was used in a study to fix the proximal tibial fracture in three foals, and follow-up revealed that the result in two of three foals was satisfying [7]. Another study conducted by Godoy et al. (2009) assessed a hybrid external fixator to fix the periarticular tibial fracture in a foal. In the mentioned study, the authors concluded that hybrid external fixators could be considered an effective surgical method to fix proximal tibial fractures [9]. One of the most secure and effective suggested surgical treatments to fix proximal physeal fractures is the use of plates and screws [1]. The use of 5.5 mm T-plates with cortical screws is recommended. They are placed in the tension surface of the bones, which is the medial aspect of the tibia [1]. Plates are of superior strength when compared to other methods and facilitate fracture reduction [6]. Moreover, due to flat physeal surface of the proximal tibial growth plate, it seems that displacement of the fractured segments in various directions may occur more commonly in the Salter-Harris type I fractures. Right-angle “L” plate, T-plate, five-hole LC-DCP, and LCPs are all recommended to fix proximal tibial physeal fractures [8]. In the present case, a seven-hole T-plate was utilized to fix the fracture. Although 5.5 mm T plate and cortical screws were suggested to be used, we have utilized a 4.5 mm T plate and cortical screws to fix the plate on the bone, and the results were satisfying. We decided to use a 4.5 mm T-plate because of the low weight of the foal, low thickness of the proximal segment of the fracture, and the availability of this plate. The major complication of the plate fixation in the medial aspect of the proximal tibia is sepsis owing to the lack of soft tissue in this region and close contact of the implant to the skin [1, 8]., A purulent fistula was observed one month following surgical procedures, which was medically treated. The infection improved but the discharge continued, so, when the fracture was deemed to be healed enough, part of the implants was removed.

In foals, tibial tuberosity fractures need to be differentiated from the normal unclosed growth plate (growth plate is apparent up to 48 months) [10]. Administration of NSAIDs, stall confinement, and controlled exercise have all been described as conservative management for tibial tuberosity fractures [10]. In a study, the success rate of conservative management of horses with tibial tuberosity fractures (without soft tissue damage) was reported to be approximately 86% [10]. In previous studies, various surgical methods were utilized to fix tibial tuberosity fractures. Gerring and Davies (1982) used a Fig. 8 tension band wire to repair tibial tuberosity fracture [11]. In another study conducted by Smith et al. cases with avulsion of tibial tuberosity were successfully treated using tension band wires and plate in combination with lag screw [12]. Furthermore, in some previous studies, the animals with tibial tuberosity fractures were treated by removing the fracture segment. In the present report, a tibial tuberosity fracture was detected on the second day of referral before surgery. Owing to the proximal and cranial dislocation of the tibial tuberosity, the fracture was confirmed.

To our knowledge, it is the first report of Salter-Harris type I fracture in proximal tibia accompanied by tibial tuberosity fracture in foal treated with a combination of a T-plate and a lag-fashion cortical screw. Overall, in agreement with previous studies [1, 6], placement of a T plate in Salter-Harris type I of proximal physeal fracture of tibia is a valuable technique to fix this type of fracture and can be recommended as an effective technique to treat Salter-Harris type I fracture in foals.

## Supplementary Information

**Additional file 1.** This file (MP4) shows the good weight-bearing on the left pelvic limb two days following surgical procedures.

## Data Availability

All data generated or analyzed during this study are included in this published article.

## Abbreviations

LC-DCP: Limited Contact Dynamic Compression Plate
LCP: Locking Compression Plate
NSAIDs: Non-steroidal anti-inflammatory drugs
